# CT-guided radioactive ^125^I seeds brachytherapy for lung oligometastases from colorectal cancer: initial results

**DOI:** 10.1186/s12885-024-12013-2

**Published:** 2024-02-26

**Authors:** Mengyao Song, Xueliang Zhou, Rongna Hou, Milan Sigdel, Yiming Liu, Chengzhi Zhang, Kaihao Xu, Xinwei Han, Dechao Jiao

**Affiliations:** Department of Interventional Radiology, The First Affiliated Hospital of Zhengzhou University, No. 1 Jianshe East Road, Zhengzhou, 450052 China

**Keywords:** Lung oligometastases, Colorectal cancer, ^125^I brachytherapy, Clinical study

## Abstract

**Objectives:**

To evaluate the safety and effectiveness of computed tomography (CT)-guided radioactive ^125^I seeds brachytherapy (RISB) for lung oligometastases (LO) from colorectal cancer (CRC).

**Methods:**

Data for 144 LOs from 70 CRC patients who underwent CT-guided RISB were retrospectively analyzed. The primary endpoints were progression-free survival (PFS) and overall survival (OS), and the secondary endpoints were technical success, local control rate (LCR), and complications. Kaplan–Meier method was used for survival analysis. Cox model was used to identify the independent predictors of poor prognosis.

**Results:**

The RISB procedures were successfully performed in all patients, and the success rate was 100%. The median follow-up was 27.8 months. The median PFS was 10.0 months (95% CI: 8.9–11.1) and the 1- and 2-year PFS rates were 32.9% and 5.9%, respectively. On multivariate analysis, serum carcinoembryonic antigen (CEA) ≤ 15 ng/ml (*P* = 0.048), middle-high differentiated pathological classification (*P* = 0.015), primary TNM stages I-III (*P* = 0.001), LO number ≤ 2 (*P* < 0.001) and cumulative gross tumor volume (GTV) ≤ 40 cm^3^ (*P* < 0.001) showed superior PFS. The median OS was 30.8 months (95% *CI*: 27.1–34.4) and the 1-, 2-, and 3-year OS rates were 95.7%, 67.4%, and 42.5%, respectively. On multivariate analysis, serum CEA ≤ 15 ng/ml (*P* = 0.004), middle-high differentiated pathological classification (*P* < 0.001), primary TNM stages I-III (*P* < 0.001), LO number ≤ 2 (*P* < 0.001), cumulative GTV ≤ 40 cm^3^ (*P* < 0.001) and system treatments combined with chemotherapy and target therapy (*P* < 0.001) showed superior OS. The LCR for 3, 6, and 12 months was 97.9%, 91.0%, and 83.6%, respectively. There were 4 cases of pneumothorax at 5.7% that required drainage.

**Conclusions:**

RISB for LO from CRC is safe and effective, and serum CEA, TNM stage, LO number, cumulative GTV, and system treatments should be emphasized for long OS.

## Key points

**•** CT-guided RISB is a new treatment for lung oligometastases from CRC.

**•** RISB can be an alternative therapy for patients who are unsuitable for surgery, ablation, or SBRT.

**•** Serum CEA, TNM stage, LO number, cumulative GTV, and system treatments are prognostic factors.

**•** RISB is a safe method with a curative effect.

## Introduction

Colorectal cancer (CRC) ranks fourth in the incidence rate of malignant tumors in China and has become one of the most important killers threatening people’s life [1]. About 50–60% of CRC patients have metastasis when diagnosed, and liver and lung are the most common sites of metastasis [2]. There is an oligometastatic state (no more than 3 metastatic organs and 5 metastatic lesions) between local tumor progression and extensive metastasis, which concept was expressed by Hellman and Weichsel Baum in 1995 [3], who emphasized the role of local treatments such as surgery, percutaneous ablation, and stereotactic body radiotherapy (SBRT) on long-term survival benefits.

Surgery is the standard treatment for resectable oligometastases from CRC, and 5-year overall survival (OS) can reach 30–60% after surgery resection of liver oligometastases, and it can reach 25–35% for patients with lung oligometastases (LOs) in previous studies [4, 5]. However, it may not be suitable for patients with contraindications such as general anesthesia problems, low performance status, serious concomitant diseases, and subjective refusal. Non-surgical local treatment strategies, represented by SBRT and ablation, are gradually becoming the preferred choice for these patients. Ablation is safe, effective, repeatable, and low in cost, which provides good curative results on peripheral LO. However, it is challenging for LO closed to the main bronchus, large vessels, or pericardium and may result in thermal damage. SBRT is characterized by the irradiation of a relatively small target with a high single dose and few fractions to achieve an equivalent biological dose to the tumor, which is similar to or even higher than conventional segmentation irradiation and has a steep dose fallout on surrounding healthy tissues. A recent meta-analysis based on 943 patients with 1290 oligometastases from 21 clinical studies concluded that 1-year progression-free survival (PFS) and OS were 51.4% (95% CI: 42.7–60.1%) and 85.4% (95% CI: 77.1–92.0%), respectively [6]. But SBRT also has its own limitations, such as the high potential for radiation-induced pneumonitis and pulmonary fibrosis that cannot be repeated in the short term [7], the equipment shortage at most Chinese hospitals because of the high price, the insufficient number of professionals because of the high requirement for tumor localization and implementation, and the difficulty paying for patients from low-income families.

Radioactive ^125^I seeds brachytherapy (RISB) was a new minimally invasive method in which the ^125^I seeds were implanted into the target lesion under the guidance of medical imaging [such as Ultrasound, Computer Tomography (CT)] and brachytherapy treatment planning system (BT-TPS). ^125^I seeds, a brachytherapy nuclide with low energy and a moderate half-life (59.6 days), can continuously emit a low dose of X and γ-rays and gradually accumulate in the tumor tissue. This highly conformal brachytherapy can ensure accurate attack on the tumor while simultaneously protecting the surrounding normal tissues and organs at risk (OAR) to the greatest extent. RISB has been widely used in the local treatment of prostate cancer [8], lung cancer [9], pancreatic cancer [10], esophageal cancer [11], biliary cancer [12] and so on. What’s more, RISB for prostate cancer and brachytherapy stent loaded with ^125^I seeds for malignant esophageal obstruction is recommended by some guidelines from the European Endoscopic Society and the Chinese Society for Esophageal Cancer Radiotherapy [13, 14]. Compared with SBRT, both can achieve high dose radiation to tumors and minimize radiation injury to the surrounding normal tissue, but RISB has its own advantages [15, 16]: (1) RISB can be widely applied under conventional CT without expensive SBRT equipment additionally; (2) all patients can complete RISB with warm home-care without frequent hospital visits; (3) RISB is cheaper for patients to reduce economic burden; (4) continuous low dose radiation is in accordance with radiotherapy “4R theory” such as repair of radiation damage, redistribution within cell cycle, reoxygenation of tumors, and repopulation of cells in tissue; (5) the local control rate (LCR) of lung tumors after RISB can be as high as 80%–96% in recent Meta analysis [17], causing relatively slighter radiation-induced lung injury that can be repeated in the short term [7], which gradually attracted the attention of radiotherapists, interventionists, oncologists and nuclide specialists. So can RISB achieve comparable efficacy to SBRT in LO from CRC? There were no related reports in the previous clinical study, as far as we know. This retrospective study preliminarily evaluated the clinical efficacy of RISB for LO from CRC, and possible influencing factors on PFS and OS were also analyzed.

## Materials and methods

### Patient selection

**Fig. 1:**
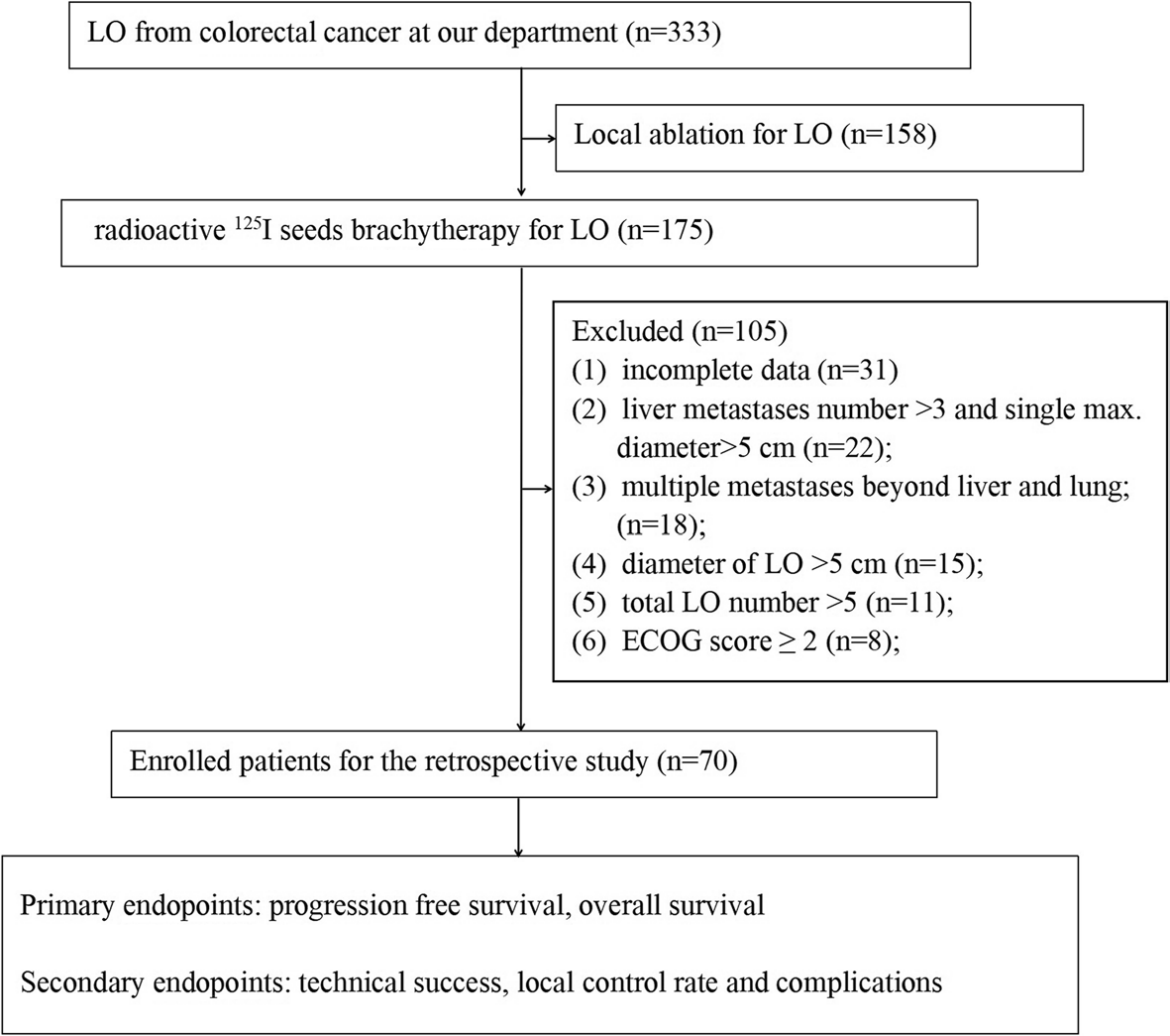
The workflow for 70 patients

This retrospective study was approved by the Ethics Committee of the First Affiliated Hospital of Zhengzhou University (Ethical Review Number: 2021-KY-400), and all data were collected from the hospital electronic information system. The inclusion criteria were as follows: (1) age range 18–75 years old; (2) radical surgical resection of primary CRC; (3) metastasis involved only lung and liver; (6) total metastases number ≤ 5; (4) limited liver metastases (metastases number ≤ 3 and max. Diameter ≤ 5 cm) are completely controlled by surgery or local ablation; (7) max. Diameter of singel LO ≤ 5 cm; (8) no lymph node metastasis previous RISB; and (9) Eastern Cooperative Oncology Group (ECOG) performance status ≤ 1. The exclusion criteria were as follows: (1) metastasis not limited to liver and lungs before RISB; (2) total metastases number > 5; (3) diameter of LO > 5 cm; (4) previous history of radiotherapy for thoracic tumor; (5) insufficient cardiovascular, hepatic, and renal function; (6) complicated with severe coagulation dysfunction (platelet count < 50 × 10^9^/L and prothrombin time > 21 s); (7) life expectancy ≤ 6 months; and (8) incomplete data. The final diagnosis of LO depended on biopsy pathology (*n* = 27) or medical image evidence (*n* = 43), and the selection process of 70 patients is presented in Fig. 1.

### ^125^I seeds and TPS

The size of ^125^I seed was (Type-6711, cylindrical, Tianjin Saide Biopharmaceutical Co., Ltd., China) 0.8 mm in diameter and 4.5 mm in length with titanium capsules, and seed radioactivity was 0.6–0.8 mCi (22.2–29.6 MBq) with a half-life of 59.6 days, which mainly emits low-dose γ-rays (35.5 keV) and soft X-rays (28.6 keV). The valid anticancer radius was 1.7 cm, and more than 90% of the barchytherapy dose was gradually released within ten months. All patients underwent pretreatment TPS (Beijing Atom & High Technical Industries Inc., China) by a clinician and physiologist (Zhou X, who had 8 years of experience) according to enhanced chest CT (lung width: 1000 HU, lung level: − 650 HU, and 5 mm slice thickness) data. With reference to International Commission on Radiation Units and Measurements (ICRU) report (No.58), to acquire the clinical target volume (CTV), the gross tumor volume (GTV) should expand by 5 mm in all directions; however, unless there is external invasion, the CTV should not exceed the anatomical limit. The prescription dose (PD) is recommended to cover the CTV with 120 Gy according to American Brachytherapy Society recommendations for prostate cancer [18] and our prior clinical experience [19]. Organs at risk (OAR) are defined as important or irradiation-sensitive organs such as spinal cord, heart or major blood vessels.

### RISB procedure

**Fig. 2:**
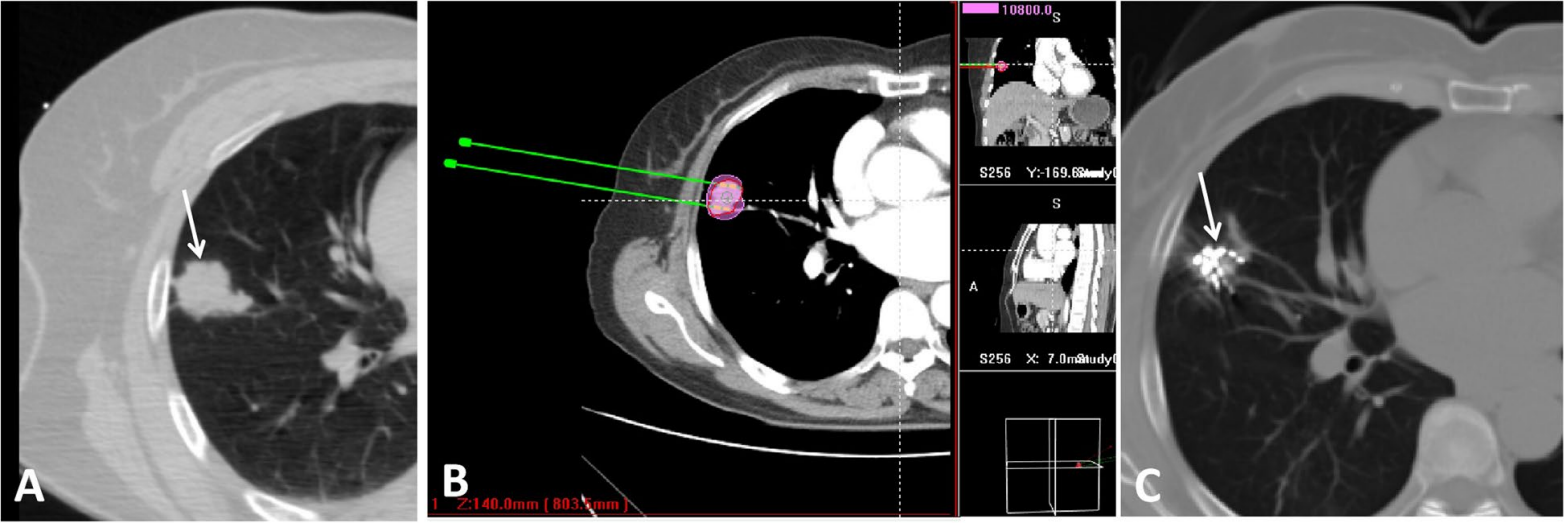
**A** 62 female patient who had a right lung oligometastase (white arrow, diameter: 3.1 cm) after radical surgical resection of primary rectal carcinoma and local ablation for one liver metastase. **B** Treatment planning system was done before radioacitve seed implantation with 120Gy prescription dose; **C** Local tumor disapeared after two months^125^I brachytherapy (white arrow)

Two experienced doctors (DC Jiao and XW Han, with 15 and 22 years of experience, respectively) performed all RISB procedures. The patient’s position was determined according to the LO location to facilitate puncture (The puncture approaches should avoid the lung vessels as much as possible). After local disinfection of the puncture point and towel laying, 2% lidocaine (5 ml) was used to achieve satisfactory local anesthesia. An 18 G puncture needle (15 cm in length, Plastic hub, Hakko Co. LTD, Japan) was inserted into the farthest end of the LO under CT guidance. Then the seed implatation gun (Tianjin Saide Biopharmaceutical Co. Ltd. China) was attached to the inserted seed needles, and the seeds were implanted from the distal to the proximal with an interval distance of 0.5–0.8 mm. Multiple needles were used at equal intervals in parallel to achieve uniform ^125^I seeds distribution when the transverse diameter of the tumor was larger than 1.5 cm according to pretreatment TPS. Finally, another CT was performed to exclude puncture-related complications, and dose verification was performed again on TPS to calculate the D90 (cumulative dose absorbed by 90% GTV) and OAR doses (Fig. 2).

### Definition and follow-up

Technical success was defined as the successful completion of RISB. Cumulative GTV was calculated by summing individual GTV for patients with multiple lesions. Progression disease (PD) was defined as any tumor recurrence or metastasis after RISB, including modality. Local failure was defined as an increase in tumor size during follow-up or the development of a new lesion in the ^125^I seed brachytherapy field. Regional failure was defined as a new lesion’s development outside the brachythrapy field in the same organ, and distant metastasis was defined as a new lesion beyond the organ. PFS was defined as the time from the start of RISB to the first documented date of PD or to the last follow-up visit. OS was defined as the time from the start of RISB to the date of death or the last follow-up visit. Local control rate (LCR) will be conducted according to the evaluation standard for solid tumor efficacy (RECIST 1.1) [20]. LCR = [complete response (CR) + partial response (PR) + stable disease (SD)]/total number of lesions × 100%. Adverse reactions were evaluated according to the criteria for common adverse events (CTCAE 4.03) of the National Institutes of Health (NIH) [21]. All patients were followed up every month during the first three months and then every 2–3 months thereafter. The contents of follow-up included routine blood tests, liver and kidney function, serum CEA, and enhanced CT.

### Statistical analysis

SPSS 19.0 software (SPSS, Inc., Chicago, IL) was used to perform all statistical analyses. All continuous data are expressed as the mean ± standard deviation (SD). PFS and OS were calculated and compared by the Kaplan–Meier method and log-rank test, and all possible variables were finally analyzed by multivariate analysis (Cox hazards model) to identify independent prognostic factors. *P* < 0.05 was considered statistically significant.

## Results

### Study population

From October 2012 to January 2019, 70 patients [mean age: (57.5 ± 7.6) years old, (range: 41–71); male/female = 37:33, 144 LO with mean GTV (31.2 ± 18.1) cm^3^] with CRC met the inclusion criteria, whose data were collected by electronic information system. Of all 70 patients, the primary CRCs (colon cancer = 34 and rectum = 36) were completely controlled by surgery, and 44 patients had limited liver metastases (metastases number ≤ 3 and max. Diameter ≤ 5 cm) that were completely controlled by surgery (*n* = 14) or local ablation (*n* = 30). There were 1, 2, 3, 4, and 5 LO in 26 (37.1%), 21 (30.0%), 17 (24.3%), 5 (7.1%) and 1 (1.4%) patients, respectively. Primary TNM stage I-III and IV were in 47 (67.1%) and 23 (32.9%) patients, and low and middle-high differentiated pathological classification were in 28 (40.0%) and 42 (60.0%) patients, respectively. The serum CEA was (28.7 ± 21.8) ng/ml (range 3.60–108.20). The total implanted ^125^I seeds were 3089, and the implanted ^125^I number per patient was (44.1 ± 16.1) (range 20.0–95.0). According to postoperative TPS evaluation, the D90 was (116.4 ± 11.8) Gy (range 92.5–144.6). As for systemic treatments, 43 (61.4%) and 27 (38.6%) accepted chemotherapy combined target therapy and chemotherapy alone, respectively, and other detailed information is presented in Table 1, and the chemothrapy regimen was capecitabine plus oxaliplatin.

**Table 1:** Basic characteristics

Characteristics	Value (%)
Total patients/LO number	70/144
Sex(male/female)	37(52.9%)/33(47.1%)
Age (mean ± SD, years old)	57.5 ± 7.6
BMI (mean ± SD, mg/m^2^)	24.6 ± 2.9
Serum CEA (mean ± SD, ng/ml)	28.7 ± 21.8
Serum CEA stratification (≤ 15 ng/ml/ > 15 ng/ml)	32(45.7%)/38(54.3%)
Primary tumor site (colon/rectum)	34(48.6%)/36(51.4%)
Pathological classification (low/middle- high differentiated)	28(40.0%)/42(60.0%)
TNM stage (stage I-III/stage IV)	47(67.1%)/23(32.9%)
ECOG score (0/1)	35(50.0%)/35(50.0%)
Number of LO per person(1/2/3/4/5 LO)	26(37.1%)/21(30.0%)/17(24.3%)/5(7.1%)1(1.4%)
Mean number of LO per person ( mean ± SD)	2.1 ± 1.0
Number of LO stratification (≤ 2 / > 2 nodules)	47(67.1%)/23(32.9%)
LO location (left/right lung)	79(54.9%)/65(45.1%)
Mean GTV per LO ( mean ± SD, *cm*^*3*^)	31.2 ± 18.1
Cumulative GTV per person ( *cm*^*3*^)	53.0 ± 22.4
Cumulative GTV stratification (≤ 40 / > 40 *cm*^*3*^)	31(44.3%)/39(55.7%)
D90 per LO (mean ± SD, Gy)	116.4 ± 11.8
Systemic treatments (C + TT/C alone)	43(61.4%)/27(38.6%)
Progression disease (Yes/no)	64(91.4%)/6(8.6%)
Disease-free interval (months)	11.0 ± 6.9
Disease-free interval stratification (≤ 12 / > 12 months)	47(67.1%)/23(32.9%)
Disease progression type (local/regional/distance failure)	9/22/61
PFS (median, 95% CI) (months)	10.0(95% CI: 8.9–11.1)
6-, 12-, 24-month PFS rates (%)	72.9%/32.9%/5.9%
OS (median, 95% CI) (months)	30.8(95% CI: 27.1–34.4)
1-, 2-, 3-year OS rates (%)	95.7%/67.4%/42.5%

*LO* Lung oligometastases, *ECOG* Eastern Cooperative Oncology Group, *PFS* progression free survival, *OS* overall survival, *CEA* Carcinoembryonic antigen, *C* Chemotherapy (capecitabine plus oxaliplatin), *TT* target therapy, *GTV* gross tumor volume

### Local control and time to progression

**Fig. 3:**
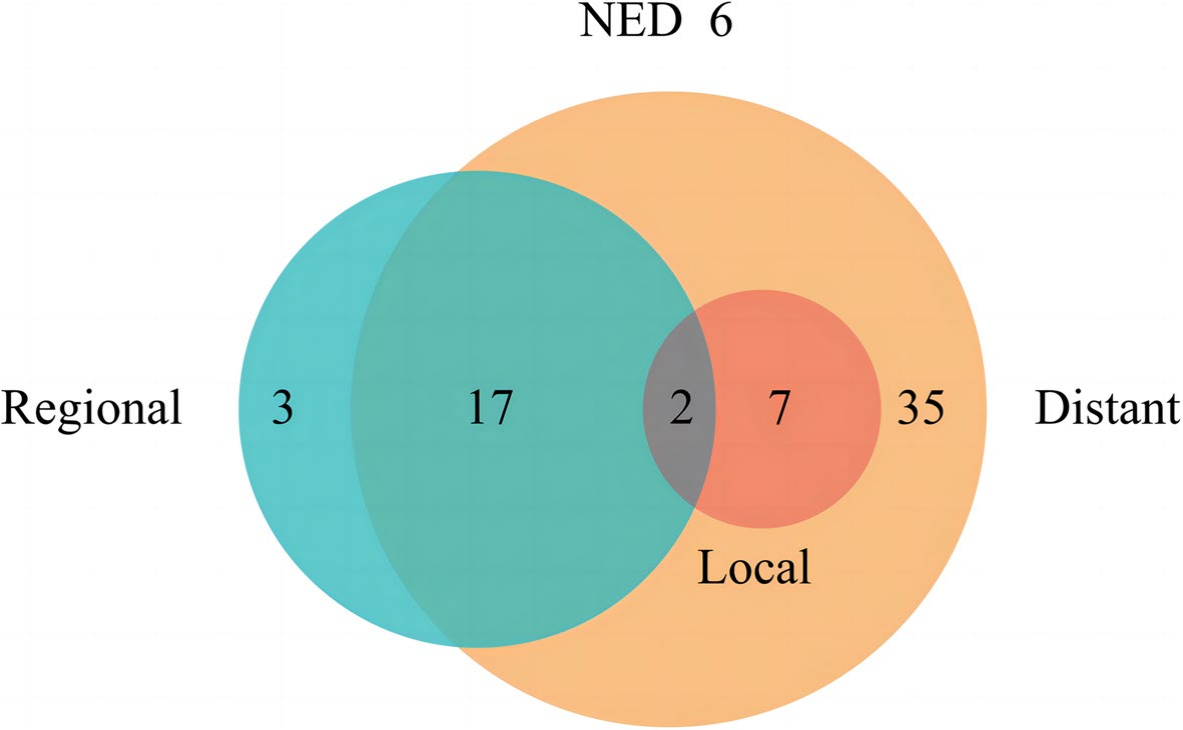
The patterns of progression disease (PD) in 64 patients: local failure (*n* = 9), regional failure (*n* = 22), distant metastasis (*n* = 61). NED: no evidence of disease progression

**Fig. 4:**
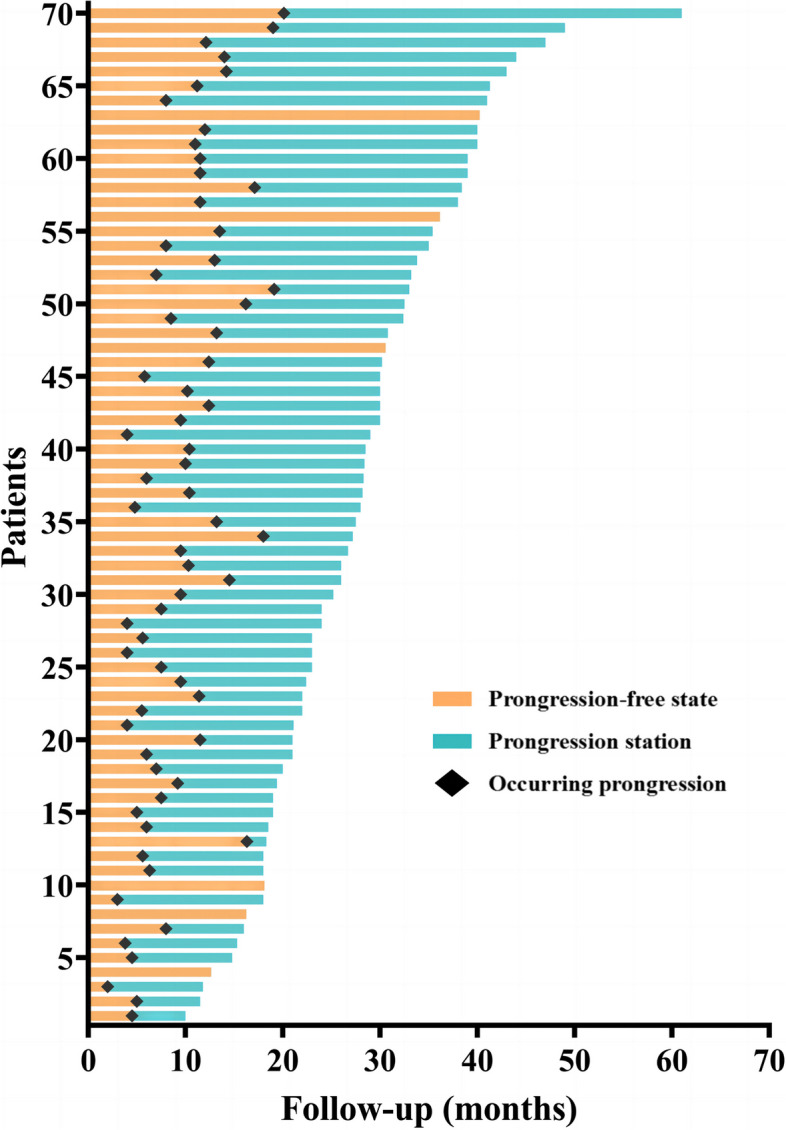
The progression free and disease progression state of all 70 patients

During the median follow-up of 27.8 months (range: 10.0–61.0), the median PFS was 10.0 months (95% CI: 8.9–11.1), and the median OS was 30.8 months (95% CI: 27.1–34.4). Technical success was achieved in 105 procedures performed to treat 144 LO, corresponding to a technical success rate of 100%. 64 (91.4%) patients experienced PD because of local failure (*n* = 9), regional failure (*n* = 22), distant metastasis (*n* = 61) (Fig. 3). The 3-, 6-, and 12-month LCR were 97.9%, 91.0% and 83.6%, respectively (Table 2). All further treatments were decided by a multidisciplinary panel composed of thoracic surgeons, respiratory physicians, oncologists, radiation physicians, pathologists, interventional radiologists and radiologists. There were 50 (time to PD ≤ 12 months) and 14 (time to PD > 12 months) patients who experienced disease progression, respectively (Fig. 4). There were 39 (55.7%) patients who experienced death. The reasons were extensive metastases (*n* = 29), cachexia (*n* = 8), acute myocardial infarction (*n* = 1), cerebral hemorrhage (*n* = 1).

**Table 2:** Local response of all 144 lesions

Months	Total LO evaluated	CR	PR	SD	PD	LCR
3 month	144	89(57.6%)	46(31.9%)	12(8.3%)	3 (%)	97.9%
6 month	144	81(56.3%)	38(26.4%)	12(8.3%)	13(9.0%)	91.0%
12 month	134	72 (50.0%)	30 (20.8%)	10 (6.9%)	22(15.3%)	83.6%

*LCR* local control rate, *CR* complete response, *PR* partial response, *SD* stable disease, *LCR* (CR + PR + SD)/total LO evaluated

### PFS and prognostic factors

**Fig. 5:**
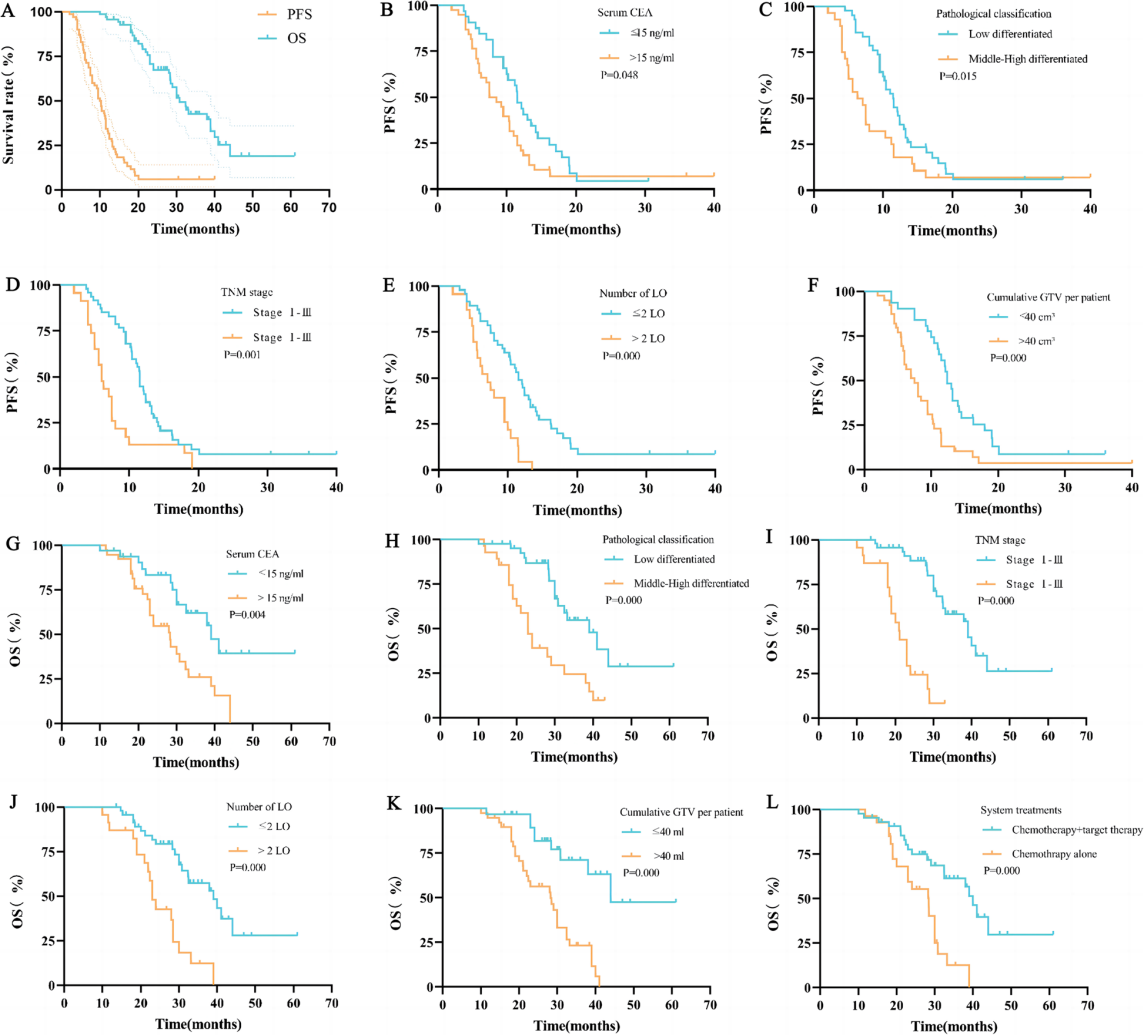
Kaplan–Meier univariate analyses of PFS and OS. **A** PFS and OS of all patients; **B** PFS of serum CEA; **C** PFS of pathological classification; **D** PFS of TNM stage; **E** PFS of LO number; **F** PFS of cumulative GTV per patient; **G** OS of serum CEA; **H** OS of pathological classification; **I** OS of TNM stage; **J** OS of LO number; **K** OS of cumulative GTV per patient; **L** OS of system treatments

The median PFS was 10.0 months (95% *CI*: 8.9–11.1), and the 1- and 2-year PFS rates were 32.9% and 5.9%, respectively. Patients with the following characteristics had superior PFS: serum CEA ≤ 15 ng/ml [11.50 months (95% *CI*: 10.26–12.75) vs 7.50 months (95% *CI*: 4.84–10.16), *P* = 0.048], middle-high differentiated pathological classification [11.40 months (95% *CI*: 9.77–13.03) vs 6.30 months (95% *CI*: 4.45–8.15), *P* = 0.015], primary TNM stage I-III [11.50 months (95% *CI*: 11.10–11.90) vs 6.00 months (95% *CI*: 4.90–7.10), *P* = 0.001], LO number ≤ 2 [11.50 months (95% *CI*: 10.02–12.98) vs 7.00 months (95% *CI*: 4.65–9.35), *P* < 0.001], cumulative GTV ≤ 40 cm^3^ [12.40 months (95% *CI*: 11.31–13.49) vs 7.50 months (95% *CI*: 5.77–9.23), *P* < 0.001] (Fig. 5). The above five factors were included in the multivariate analyses using the Cox hazards model, and serum CEA ≤ 15 ng/ml (HR: 1.906, *P* = 0.020), primary TNM stage I-III (HR: 2.749, *P* = 0.003), LO number ≤ 2 (HR: 2.150, *P* = 0.025), and cumulative GTV ≤ 40 cm^3^ (HR: 2.012, *P* = 0.033) were independent factors for superior PFS. The following characteristics were not significantly different in the univariate analysis: sex (*P* = 0.168), age (*P* = 0.790), Body Mass Index (BMI) (*P* = 0.564), smoke abuse (*P* = 0.751), primary tumor site (*P* = 0.151), ECOG score (*P* = 0.671) and system treatments (*P* = 0.356) (Tables 3 and 4).

**Table 3:** Univariate analysis of PFS and OS

Factors	PFS (months)		OS (months)	
	Median, (95% CI)	*P*	Median, (95% CI)	*P*
Sex		0.168		0.250
Male	8.00, (6.01–9.99)		30.00, (24.44–35.56)	
Female	11.50, (10.16–12.84)		38.00, (22.88–53.13)	
Age		0.790		0.530
≤ 60 years old	10.40, (1.20–8.04)		30.00, (26.35–33.65)	
> 60 years old	9.50, (6.96–12.04)		32.50, (25.86–39.14)	
Body mass index		0.564		0.345
≤ 25 kg/m^2^	8.50, (5.79–11.21)		30.00, (26.87–33.14)	
> 25 kg/m^2^	10.40, (9.28–11.52)		38.00, (25.067–50.93)	
Smoke abuse		0.751		0.133
Yes	9.50, (6.87–12.13)		30.00, (27.61–32.38)	
No	10.40, (8.64–12.16)		39.00, (27.19–50.81)	
Serum CEA		**0.048**		**0.004**
≤ 15 ng/ml	11.50, (10.26–12.75)		39.00, (30.30–47.69)	
> 15 ng/ml	7.50, (4.84–10.16)		28.30, (22.85–33.75)	
Primary tumor site		0.151		0.201
Colon	11.00, (9.29–12.71)		32.50, (29.50–35.49)	
Rectum	8.00, (5.65–10.35)		30.00, (27.90–32.10)	
Pathological classification		**0.015**		**0.000**
Low differentiated	6.30, (4.45–8.15)		23.00, (20.17–25.84)	
Middle-High differentiated	11.40, (9.77–13.03)		39.00, (30.54–47.46)	
TNM stage		**0.001**		**0.000**
Stage I-III	11.50, (11.10–11.90)		39.00, (31.95–46.05)	
Stage IV	6.00, (4.90–7.10)		21.00, (17.94–24.06)	
ECOG score		0.671		0.504
PS 0	10.40, (8.95–11.85)		32.50, (27.62–37.38)	
PS 1	9.50, (8.35–10.65)		29.00, (20.18–37.82)	
Number of LO		**0.000**		**0.000**
≤ 2 LO	11.50, (10.02–12.98)		39.00, (29.33–48.67)	
> 2 LO	7.00, (4.65–9.35)		23.00, (20.82–25.18)	
Cumulative GTV per patient		**0.000**		**0.000**
≤ 40 *cm*^*3*^	12.40, (11.31–13.49)		45.95(38.25–53.66)	
> 40 *cm*^*3*^	7.50, (5.77–9.23)		28.30, (20.74–35.86)	
System treatments		0.356		**0.000**
C + TT	11.20, (10.10–12.30)		40.00, (36.57–43.43)	
C alone	7.50, (4.32–10.68)		28.40, (22.26–34.54)	

*AFP* alpha fetoprotein, *BCLC* Barcelona Clinic Liver Cancer, *CI* Confidence interval, *ECOG* Eastern Cooperative Oncology Group, *OS* overall survival, *PFS* progression free survival, *PO* Pulmonary oligometastases, *C* Chemotherapy, *TT* target therapy

**Table 4:** Cox-regression multivariate analysis of PFS and OS

Factors	PFS (months)		OS (months)	
	HR (95% CI)	*P*-value	HR (95% CI)	*P*-value
CEA level (≤ 15 / > 15 ng/ml)	1.906(1.107–3.283)	**0.020**	2.493(1.146–5.423)	**0.021**
Pathological classification (Low/Middle-High differentiated)	1.298(0.707–2.384)	0.400	1.972(0.793–4.906)	0.144
TNM stage (stage I-III/IV)	2.749(1.412–5.351)	**0.003**	9.215(3.199–26.549)	**0.000**
Number of LO (≤ 2 / > 2 LO)	2.150(1.100–4.200)	**0.025**	1.678(0.772–3.650)	0.192
Cumulative GTV (≤ 40 / > 40 *cm*^*3*^)	2.012(1.060–3.821)	**0.033**	4.164(1.791–9.679)	**0.001**
System treatments(C + TT/C alone)	-	-	2.388(1.024–5.572)	**0.044**

*CI* Confidence interval, *HR* hazard ratio, *OS* overall survival, *PFS* progression-free survival, *LO* Lung oligometastases, *C* Chemotherapy, *TT* target therapy

### OS and prognostic factors

The median OS was 30.8 months (95% CI: 27.1–34.4), and the 1-, 2- and 3-year OS rates were 95.7%, 67.4% and 42.5%, respectively. Patients with the following characteristics had superior OS: serum CEA ≤ 15 ng/ml [39.00 months (95% CI: 30.30–47.69) vs 28.30 months (95% CI: 22.85–33.75), *P* = 0.004], middle-high differentiated pathological classification [39.00 months (95% CI: 30.54–47.46) vs 23.00 months (95% CI: 20.17–25.84), P < 0.001], primary TNM stage I-III [39.00 months (95% CI: 31.95–46.05) vs 21.00 months (95% *CI*: 17.94–24.06), *P* < 0.001], LO number ≤ 2 [39.00 months (95% CI: 29.33–48.67) vs 23.00 months (95% CI: 20.82–25.18), *P* < 0.001] and cumulative GTV ≤ 40 cm^3^ [45.95 months (95% CI: 38.25–53.66) vs 28.30 months (95% CI: 20.74–35.86), *P* < 0.001] (Fig. 5). The above six factors were included in the multivariate analyses, and serum CEA ≤ 15 ng/ml (HR: 2.493, *P* = 0.021), primary TNM stage I-III (HR: 9.215, *P* < 0.001), cumulative ≤ 40 cm^3^ (HR: 4.164, *P* = 0.001) and systemic treatments combined with chemotherapy and target therapy (HR: 2.388, *P* = 0.044), were independent factors for superior OS. The following characteristics were not significantly different in the univariate analysis:sex (*P* = 0.250), age (*P* = 0.530), BMI (*P* = 0.345), smoke abuse (*P* = 0.133), primary tumor site (*P* = 0.201), ECOG score (*P* = 0.504) (Tables 3 and 4).

### Complications

Four patients experienced pneumothorax pulmonary compression of 30%–60% during the procedure, and chest drainage was needed to relieve the symptom. All of them required 2–3 days of hospitalization, and they were evaluated as major complications. Minor complications were intrapulmonary hemorrhage (*n* = 31), a small amount of pneumothorax not requiring treatment (*n* = 11), and hemoptysis (≤ 10 ml, *n* = 3). No RISB-associated massive bleeding, irradiation pneumonitis or pulmonary fibrosis, pulmonary infection, pleural fistula, or death occurred during treatment and follow-up.

## Discussion

Approximately half of the patients developed distant organ metastases when CRC was initially diagnosed, and the most common metastatic sites include liver, lung, brain, and bone [22]. Active local treatments such as hepatectomy, ablation, and SBRT for liver metastasis have been accepted by multiple clinical guidelines, and it is believed that this strategy can significantly improve the long-term survival rate [23, 24]. However, as the second most common site of metastasis for CRC after the liver, there are few detailed guidelines focusing on CRC with lung metastases. Previous study showed that only 10% patients were suitable for radical lung metastasectomy among the initial lung metastases, for the remaining 90% of patients, non-surgical local therapy is an alternative option combined with systematic therapy [25].

Non-surgical local treatment strategies, represented by SBRT and ablation, are a valid option for patients with oligometastatic disease. SBRT is a non-invasive treatment and is able to deliver ablative radiation doses to target lesions, short of equipment limit its application at China [7, 26]. Therefore, we hypothesized that RISB may be ideal for the treatment of lung oligometastases from CRC, hoping it has a similar effect compared to conventional local therapy and can be an alternative choice when SRBT and ablation are unsuitable for specific patients. RISB has been increasingly practiced in the clinical treatment of NSCLC since the 1980s [27]. A recent meta-analysis including 15 studies and 1188 cases has systematically reported that RISB combined with chemotherapy has a higher overall response rate (RR = 1.84, 95% CI: 1.65–2.05), better OS (HR = 0.66, 95% CI: 0.50–0.86) as compared to chemotherapy alone for the treatment of NSCLC [17]. However, compared to liver oligometastases, the studies of local therapy, especially RISB, that focus on LOs from CRC are much fewer, and there are no authoritative guides that describe the therapy in detail. Its efficacy and safety remain unclear.

Kinj et al. [17] treated 53 oligometastatic patients with 87 lung lesions from CRC and reported that 1-year LCR of SBRT was 79.8%, which is also similar to the present study (1-year LCR of 83.6% at present study). Agolli et al. [28] previously reported the SBRT treatment of 44 patients and 69 lesions. The study showed 2-year OS was 67.7%, which is similar to the present study (2-year OS rate of 67.4%). A prospective study about RISB conducted by Wang et al. [29] reveals a 1-year LCR of 33.3% and a mOS of 18.8 months in 33 patients with 126 bilateral lung recurrence lesions from CRC, which is much lower than our study (79.8% and 30.8 months). This difference may be due to the fact that the inclusion criteria for their study were limited to patients with bilateral lung oligometastases and failure of standard chemotherapy in the majority of patients. The 2-year PFS reported in most of the SBRT studies were 16.2%–27% [17, 28, 30–32], which is significantly higher than that of our study ( 5.9% at present study). The reasons for the analysis are as follows: (1) RISB has high puncture skills requirement for the operator, and when the puncture cannot obtain a satisfactory ^125^I seed distribution, it means that the dose distribution is uneven (the D90 range in this study was 92.5–144.6 Gy), while SBRT has a standard operating process, controllable and repeatable dose, so the efficacy can be replicated in different centers; (2) The difference in inclusion criteria for selected cases is also an important operational aspect; (3) These studies come from different countries, and there may be differences in the efficacy of RISB and SBRT among different ethnic groups.

Numerous studies also investigated the potential variables that affect effectiveness when focusing on local therapies for lung metastases. Agolli et al. [28] reported that multiple lung metastases were significantly associated with worse PFS. Kinj et al. [17] demonstrated that there was a significant OS detriment in patients with ≥ 3 metastases and patients with a larger GTV). In a prospective study, it was reported that the degree of tumor differentiation, growth rate, and tumor size could affect local efficacy. It was also reported that serum CEA level was associated with the OS [33]. In our study, we similarly included the potential factors in survial analysis, and the results show that lower serum CEA levels (≤ 15 ng/ml), middle-high pathological differentiation, lower primary TNM stages (I-III), fewer LO numbers (≤ 2) and smaller cumulative GTV (≤ 40 cm^3^) were significantly associated with better PFS. In regards to OS, in addition to the factors mentioned above, systemic treatment combined with chemotherapy and target therapy is also a contributing factor to the superior prognosis. These results were similar to the data from the literature mentioned above and suggest which patients are more likely to benefit from RISB. Therefore, these factors should be taken into account when designing treatment strategies for patients during our clinical practice.

As for complications including hemoptysis, pneumothorax, and pneumorrhagia occurred in a significant portion of patients who underwent RISB, which is mainly due to punctures. These complications can be reduced by minimizing the number of punctures and ensuring that they are as far as possible from large vessels and trachea. In addition, it is suggested that both lungs should not be treated at the same time because of life threatening bilateral pneumothorax. No irradiation-related pneumonitis or pulmonary fibrosis occurred during the therapy period. The results proved that it has an equal effect but milder lung tissue damage compared to SBRT, which is also demonstrated at clincial study from Li et al. [26].

Our study has some limitations. This study was a retrospective, small-sample, single-center study with a median follow-up of 27.8 months. The conclusion needs to be confirmed by prospective multiple center large sample study with longer follow-up in the future. In conclusion, our study suggests that RISB for LO from CRC is safe and effective, and serum CEA, TNM stage, LO number, cumulative GTV, and system treatments should be emphasized for long OS.

## Data Availability

The datasets used or analyzed during the current study are available from the corresponding author on reasonable request.

## Abbreviations

RISB: Radioactive ^125^I seeds brachytherapy
LO: Lung oligometastases
CRC: Colorectal cancer
SBRT: Stereotactic body radiotherapy
BT-TPS: Brachytherapy treatment planning system
OAR: Organs at risk
CT: Computer tomography
CEA: Carcinoembryonic antigen
GTV: Gross tumor volume
PFS: Progression-free survival
OS: Overall survival
LCR: Local control rate
CR: Complete response
PR: Partial response
SD: Stable disease
PD: Progression disease
ECOG: Eastern Cooperative Oncology Group
NIH: National Institutes of Health
ICRU: International Commission on Radiation Units and Measurements
